# A brain machine interface framework for exploring proactive control of smart environments

**DOI:** 10.1038/s41598-024-60280-7

**Published:** 2024-05-14

**Authors:** Jan-Matthias Braun, Michael Fauth, Michael Berger, Nan-Sheng Huang, Ezequiel Simeoni, Eugenio Gaeta, Ricardo Rodrigues do Carmo, Rebeca I. García-Betances, María Teresa Arredondo Waldmeyer, Alexander Gail, Jørgen C. Larsen, Poramate Manoonpong, Christian Tetzlaff, Florentin Wörgötter

**Affiliations:** 1 SDU Applied AI and Data Science, University of Southern Denmark, 5230 Odense, Denmark; 2 Department for Computational Neuroscience, University of Göttingen, 37077 Göttingen, Germany; 3 Sensorimotor Group, German Primate Center – Leibniz-Institute for Primate Research, 37077 Göttingen, Germany; 4 Cognitive Neuroscience Laboratory, German Primate Center – Leibniz-Institute for Primate Research, 37077 Göttingen, Germany; 5 Faculty of Biology and Psychology, University of Göttingen, 37077 Göttingen, Germany; 6 Embodied AI and Neurorobotics Lab, University of Southern Denmark, 5230 Odense, Denmark; 7 Life Supporting Technologies Research Group, ETSIT, Universidad Politécnica de Madrid, 28040 Madrid, Spain; 8 Bio-inspired Robotics and Neural Engineering Lab, Vidyasirimedhi Institute of Science and Technology, Rayong, 21210 Thailand; 9 Group of Computational Synaptic Physiology, Department for Neuro- and Sensory Physiology, University Medical Center Göttingen, 37073 Göttingen, Germany

**Keywords:** Neural decoding, Quality of life, Biomedical engineering

## Abstract

Brain machine interfaces (BMIs) can substantially improve the quality of life of elderly or disabled people. However, performing complex action sequences with a BMI system is onerous because it requires issuing commands sequentially. Fundamentally different from this, we have designed a BMI system that reads out mental planning activity and issues commands in a proactive manner. To demonstrate this, we recorded brain activity from freely-moving monkeys performing an instructed task and decoded it with an energy-efficient, small and mobile field-programmable gate array hardware decoder triggering real-time action execution on smart devices. Core of this is an adaptive decoding algorithm that can compensate for the day-by-day neuronal signal fluctuations with minimal re-calibration effort. We show that open-loop planning-ahead control is possible using signals from primary and pre-motor areas leading to significant time-gain in the execution of action sequences. This novel approach provides, thus, a stepping stone towards improved and more humane control of different smart environments with mobile brain machine interfaces.

## Introduction

**Figure 1:**
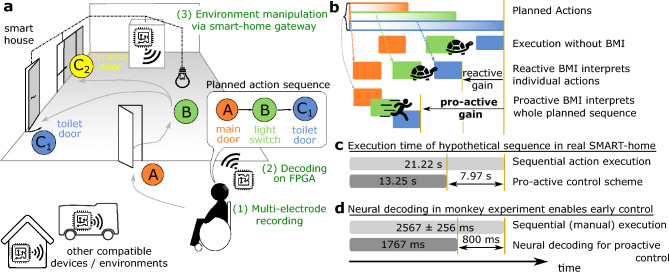
Concept of BMI-based proactive smart home control. (**a**) The use case for proactive control comprises (1) recording of multi-channel brain activity, (2) a mobile device decoding sequences of planned actions and being situated at the patient for all-day support, and (3) a smart home gateway infrastructure, through which the mobile device can interact with multiple smart environments. (**b**) Illustration of proactive time gain: action sequences are planned quickly, but their execution takes time. Reactive BMIs decode the currently planned/executed action, leading to a reactive time-gain compared to execution without BMI. Decoding the plan of a whole action sequence enables proactive BMIs, resulting in larger proactive time-gains due to faster execution of actions. (**c**) Executing the hypothetical scenario from panel (**a**) in a real smart-home environment, a proactive control approach allows significantly faster execution ($38\%$) when compared to the sequential execution of the action sequence (numbers taken from Supplementary Video SV1, see Discussion). Note that we do not compare to the time of successful decoding, which would result in an unrealistically higher gain, but include time for actuator and actor movement. (**d**) In the proof-of-principle monkey experiment considered in this study—an instructed button press after a walk-and-reach movement—the potential gain is based on the time neural recordings are decoded and can lead to a speed up by $30{ \%}$.

Brain-machine interfaces (BMIs) are tools that can support the daily life of disabled and healthy people. A BMI receives brain signals as inputs, decodes these, and outputs commands to control connected devices (Fig. 1a^1,2^). Often these are prostheses to enable actions or extend the range of motion^2^. In addition, BMIs can support users’ independence^2,3^ by allowing for complex actions or leading to faster action execution. Here, especially the coupling of BMIs with nowadays ubiquitous smart devices opens a promising field of new applications.

In daily life, actions are occurring as part of action sequences. In a house, for example, a sequence could be walking towards a door, opening it, switching on the light, etc. (grey arrows in Fig. 1a). Using a BMI interfacing with a smart house, these actions could be offloaded to smart devices connected to this environment leading to faster execution (*reactive time gain* in Fig. 1b). Yet, because current BMI systems are solely *reactive*, a user needs to explicitly instruct the multiple devices for every single action in such a sequence. To speed up and simplify action execution, we propose to use a *proactive* BMI control scheme, where planned actions further down the sequence are decoded and the actions are initiated seamlessly, that is without the need for the agent to issue individual commands (Fig. 1b). In practice, a proactive controller could be active in parallel to an established reactive controller and initiate upcoming actions ahead of time.

In this study, we explore the viability of such a proactive control scheme using a newly developed hard- and software framework for experiments with non-human primates, which is inspired by future human use cases, in which, for example, disabled users employ proactive BMIs to interact more efficiently with smart devices in their surrounding (Fig. 1a, b).

We show in a proof-of-concept that the time saved through proactive control—the *proactive time gain*—can be substantial in comparison to a reactive system, depending on how long the individual actions actually are (Fig. 1b, see Fig. 1c for a hypothetical smart house application and Fig. 1d for the potential gain in the monkey experiments), which would result in a noticeable improvement for the BMI user.

To make proactive control possible, action plans must be decodable from brain signals prior to action execution. Currently, the majority of BMI applications use electroencephalography (EEG). While non-invasive and harmless, surface EEG has a low spatial resolution and current attempts to decode action plans from EEG signals achieve, thus, rather low accuracy^4,5^. Intracranial electrode arrays, implanted in the task-relevant brain regions and recording from individual cells, could solve this problem^6^. Interestingly, information of upcoming actions can be extracted from brain areas that are typically accessed by motor BMIs, such as primary motor cortex M1, the parietal reach region^7^ or the dorsal premotor cortex^8,9^, and recent findings indicate that they might carry planning information on whole action sequences^10,11^.

However, stable long-term recordings from intracranial electrode arrays are difficult to obtain such that the performance of a fixed decoder degrades substantially over time^12–14^, requiring a regular re-calibration of the decoder. A widely used approach for re-calibration is to continuously correct the decoder for incremental changes based on data of the user’s behaviour and brain activity^15–19^. However, in daily life, a continuous monitoring of the user cannot be guaranteed and irregular breaks for days or even weeks have to be taken into account. To cope with this, a recently developed approach—so-called manifold realignment—exploits the inherent correlations of neuronal activities. These correlations imply that the relevant information is encoded on a low-dimensional latent manifold of the high dimensional neural activity space. A pioneer study^14^ has shown that, while the high-dimensional activity space could change, this manifold is stable over longer time periods enabling the re-calibration of a BMI-decoder by aligning the data of different recording sessions in the low-dimensional space. However, it remains unclear whether the online re-calibration of a decoder by manifold realignment requires fewer calibration trials than needed for training a new decoder.

**Figure 2:**
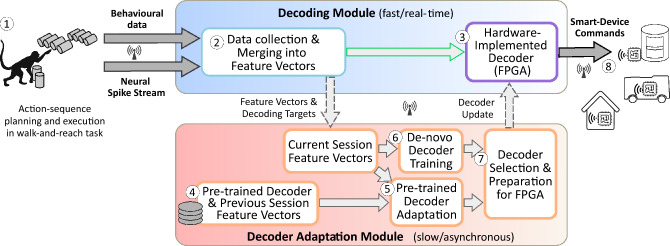
Proactive control framework presented in this study. We employ a walk-and-reach task, in which freely moving monkeys perform action sequence planning and execution in a smart cage that allows for tracking behaviour and neural activities^11^. For proactive control, brain activities during sequence planning are decoded by a fast decoding module implemented on embedded hardware. In a proof-of-concept setting, the decoding results are then transformed into control commands for smart appliances outside the smart cage. To cope with recording instabilities, the framework also comprises a decoder adaptation module, which utilizes the adaptation of pre-trained decoders from previous sessions to render the BMI functional despite breaks for days or weeks.

**Figure 3:**
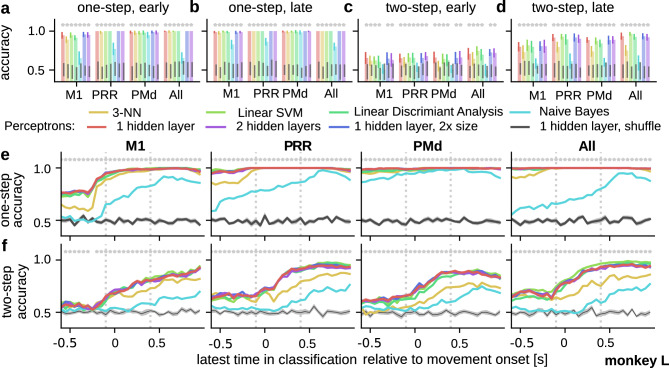
Offline decoding of planned action sequences from neural activities. Comparison of the classification accuracy of various decoding algorithms for predicting the upcoming action in an one-step action sequence (close reach targets) before (**a**) and after (**b**) movement onset. Pale coloured bars and solid coloured error bars mark mean and standard deviation from a four times repeated, five-fold, stratified cross-validation for each classifier. Grey bars mark standard deviations for the same procedure with shuffled targets. Asterisks indicate significant difference (one-sided Mann–Whitney U-Test). Classifications were based on data from individual recordings from M1, PMd, PRR or from pooled activity (x-axis). (**c, d**) Same as a and b, but for decoding the last action of a two-step action sequence. (**e**) Decoding accuracy for an upcoming action (one step ahead in the sequence) by different classifiers for a $800\,\text {ms}$ moving window ending at the time indicated on the x-axis. Classifications were based on data from individual recordings from M1 (1st panel), PRR (2nd panel), PMd (3rd panel) or from pooled data (4th panel). Grey curve represents the accuracy of the one-layer perceptron classifier with shuffled targets and asterisks a significant difference between shuffled and non-shuffled condition (Mann–Whitney U-Test). Shaded areas mark one SEM for the respective curves. Grey vertical lines mark the time points shown in (**a/c**) and (**b/d**). (**f**) Same as e, but for decoding the action plan of a two-step action sequence. All depicted results are obtained from monkey L. Comparable results from a second monkey are found in Figure S7 in the Supplemental Information.

Here, we present the vision of a new, proactive BMI-technology together with a framework for proactive control that builds on our technology for reliable decoding plans of action sequences. The decoding of predictive planning information provides the basis for the proactive control of smart devices. Hereby, manifold realignment is a core component that provides a highly efficient and robust way for re-calibrating the decoder between sessions being several days apart. As this technology is still at an experimental stage, we implemented an open-loop proactive control framework (Fig. 2) based on a setup with recordings from experimental animals (rhesus monkeys, Fig. 2, ①). This comprises a wireless neural data recording system (Fig. 2, ②) coupled to a field-programmable gate array (FPGA)-embedded decoder (Fig. 2, ③), allowing a low-power, battery-based, on-patient, mobile usage of our technology in the future. The FPGA links to a secure smart home gateway^20^ to issue commands for smart devices (Fig. 2, ⑧), allowing future users to proactively control different smart environments securely over open networks. In addition, the decoder adaption framework (Fig. 2, bottom, ④ to ⑦) allows the re-calibration of the decoder from previous sessions to the current data based on the technique of manifold realignment. This allows for fast adaptation of the decoder on a daily basis, even after long breaks. Hence, a comprehensive and fast-responding framework results, which can be transferred to humans under the condition that clinically approved recording systems become available.

## Results

### Neural recording and task design

Using intracranial floating microelectrode arrays, recordings were made from the primary motor cortex (M1), the dorsal premotor cortex (PMd), and the parietal reach region (PRR) in trained rhesus monkeys that are freely moving in a specially designed smart-cage^11^—an environment that can provide visual cues through programmable LED lamps and tracks monkey behaviour in real-time through touch sensors (Fig. 2, ①; see^11^ for details). The task for the monkey is to either immediately reach for or to walk and reach for a target. The monkey is instructed about the target by one of the LED lamps and, after the end of a variable waiting period indicated by a visual go-cue, starts to move towards it, and touches the indicated lamp. We consider trials with an immediate reach target as one-step actions, while trials consisting of (1) a walking-towards-the-target action that is being followed by (2) a touching action, are two-step action sequences. This task design has already been successfully employed to demonstrate that the recorded motor areas (PMd, PRR) encode planning information^11^. The recorded spike- as well as behavioural data are then merged and collected into feature and target vectors for decoding (Fig. 2, ②, see Methods for details).

In the next sections we will employ offline analyses to (a) quantify the strength of planning-encoding neuronal activity in different brain areas and (b) to design a compact real-time compatible method for decoding this activity in spite of the day-by-day variability of neuronal recordings. In the later sections, we will then apply this to create a framework for proactive online BMIs which we will test under real-time conditions in an open-loop setting.

### Offline verification of planning information in unsorted neural activity

A user-friendly BMI discourages the application of commonly used spike-sorting algorithms that need to be tuned by trained experts in every session. Thus, as a first step to develop a decoder for our BMI framework (Fig. 2, ③), we tested whether planning information can also be obtained from unsorted spike-data.

To this end, we evaluated the capability of a broad variety of commonly used classification algorithms to decode the final reach target for one-step and two-step action sequences at different time points (see Methods section). We chose algorithms with few parameters only to keep the approach compatible with an embedded system implementation.

Upcoming actions can be decoded well at time points both before (Fig. 3a) and after movement onset (Fig. 3b). The recordings from PMd and PRR as well as the joint data from all electrodes provide high decoding accuracy, whereas the accuracy from M1 tends to be lower. Most of the tested decoders yield similar decoding accuracies. Only the classifiers that make assumptions about the data—as for example the naïve Bayesian classifier (independent features) or the nearest neighbor classifier (each feature has equal influence)—yield a lower accuracy. Similarly, the results for decoding a two-step sequence are comparable for most classification algorithms (Fig. 3c, d), but show lower accuracy than the one-step-action decoding.

The similarity of the results across decoding algorithms, however, indicates that the reduced accuracy is inherent to the recorded brain activities rather than the decoding method. Also, as most classifiers yielded similar results in the offline analysis, our BMI framework is based on the perceptron with two hidden layers, as it consists of individual processing units that can be efficiently parallelised and accelerated in hardware and the training benefits from recent advances in deep neural networks.

For the two-step sequences, decoding accuracy in all brain areas is above chance level before movement onset (Fig. 3c) and rises to around 80% shortly after the movement has begun (Fig. 3d). By analyzing the decoding accuracy as a function of time relative to movement onset, we show that for individual upcoming actions (one-step, Fig. 3e) only the decoder performance from M1 shows a rise shortly before movement onset. By contrast, for decoding two-step sequences all accuracy curves (Fig. 3f) show a significant rise around movement onset. Thus, an earlier decoding, that would provide a higher proactive gain, leads to a lower accuracy implementing a trade-off between decoding performance and proactive gain in our BMI use case. This trade-off is inherent to the recorded brain activities (compare^21^) and needs to be considered by the user according to their use case.

In summary, decoding of planning activity, both for one and two step sequences, is indeed possible from unsorted spike data. Yet, these results are based on analyses from single recording sessions. Hence, the here-analysed decoders would need additional modifications to become capable of dealing with the above described recording instabilities that arise from one day to the next in future patient use.

### Offline analyses to develop a fast online decoder re-calibration method

**Figure 4:**
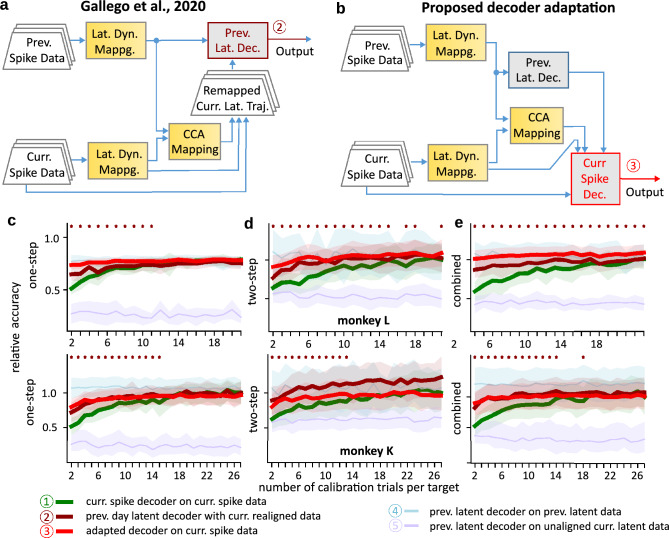
Decoder re-calibration by manifold realignment. (**a**) Workflow for using manifold realignment to project the current day data into the previous day latent manifold in order to use a pre-trained classifier as proposed by^14^. Latent manifolds and trajectories are determined for data from both, current and previous recording sessions (lat. dyn. mappg.). Then CCA is used to obtain an alignment between the latent trajectories (CCA mapping). Using this alignment, current day data can be projected into the previous day latent manifold (remapped curr. lat. traj.) and a decoder trained on previous latent trajectories (prev. lat. dec) can be applied. (**b**) Workflow for using manifold realignment to adapt pre-trained classifiers in order to apply them *directly* to current day neural spiking activities. Here, instead of transforming the current day data, the decoder pre-trained on previous day latent trajectories is adapted using the manifold projection and alignment transformations. The so-adapted decoder (curr. spike dec.) can be directly applied to current day spike data and is, thus, ideally suited for embedded hardware implementation. (**c**) Comparison of the accuracy for one-step action plan decoding against the number of available trials per reach target in a new recording session. Results are shown relative to the accuracy of a de-novo decoder on the full data set. Solid curves and shaded regions depict mean and standard deviation for a new, de-novo decoder (green), a pre-trained decoder working on realigned data (dark red, as in **a**) and an adapted pre-trained decoder by aligning latent neural trajectories (light red as in **b**). Asterisks mark a significant difference between de-novo and adapted decoder (Mann–Whitney-Test). As a control, the accuracy of the pre-trained decoder on its training data (light blue) as well as on the unaligned latent trajectories from the new recording session (light purple) are shown, too. Top and bottom panel display results from two different monkeys. (**d**) Same for two-step sequence plans (4 possible targets). (**e**) Same for combined data set with one- and two-step plans and decoders trained on all eight targets.

Thus, as a second step, we developed a decoder adaptation mechanism (Fig. 2 bottom, ④ to ⑦), which deals with these instabilities. In particular, we aimed at obtaining a decoder that provides good accuracy within an ongoing (current) recording session after as few “calibration” trials as possible. Here, “calibration trials” would be trials at the beginning of a session, where the proactive controller is not yet active and information on the plan needs to be provided to train or adapt the decoder. Note that in our use-case, this information is easily obtained from the smart environment while action sequences are executed.

In order to avoid training a new decoder for every session, we adapted a decoder pre-trained during a previous experimental session to work with current recordings. For this, we built on the finding that neural activities in the motor cortices are typically constrained to a low-dimensional manifold (see^22^ for a review). The latent trajectories in this manifold are stable over time, such that the manifolds from two different time points can be aligned to each other even when the recordings are months apart^14^. For linear manifolds, this alignment (see Fig. 4a) can be done by canonical correlation analysis (CCA)—a technique that provides a linear mapping through which trial-matched data from two manifolds become maximally correlated. The alignment also enables the use of pre-trained decoders from previous sessions on data from another recording session^14^.

We asked whether such alignment of pre-trained decoders provides good decoding with fewer “calibration” trials than needed when training a new decoder from scratch. To test this, we used recordings from two different days, but selected only a subset from the second day (that is, the “calibration” trials) with a given number of trials for each reach target. For comparison, we then trained a new decoder, which we will call “de-novo” decoder in the following, on this limited data set and evaluated its decoding performance on the rest of the second day recordings. Thus, this de-novo decoder corresponds to the ones evaluated in Fig. 3, but is based on fewer trials. All accuracies are shown relative to the accuracy reached by a de-novo decoder on the full data set. For single action prediction, we find that decoding accuracy rises only slowly reaching saturation after around 12 trials per class, which amounts to 48 training trials (for 4 possible targets, Fig. 4c, green curve). For the two-step sequences or a combined data set of one- and two-step sequences, the gain in decoding performance is even slower and reaches saturation only after 20 trials per class (Fig. 4d, e, green curve). This would amount to an immense calibration effort for the BMI user and could severely impact its acceptance level.

Thus, we next tested decoders with manifold alignment. To this end, we determined the low-dimensional manifolds for each data set as the first 50 components of a principal component analysis (PCA; Fig. 4a, b, “Lat. Dyn. Mappg.”) on the available data. From this, we obtained the latent trajectories by projecting the data to this hyperspace and performed CCA to get linear transformation matrices that align the two manifolds (CCA Mapping in Fig. 4a, b). We then obtained a pre-trained decoder by training another multi-layer perceptron on the latent trajectories from the full previous day data set (Fig. 4a, dark red box). Analogous to^14^, we used the transformation matrices to project the current day latent trajectories into the manifold of the previous day (Fig. 4a, “Remapped curr. lat. traj.”) and applied the pre-trained decoder to the new data. We evaluated the performance of this decoder and found that the decoding accuracy rises much faster and reaches its saturation after only two trials per target for the single action (8 trials, Fig. 4c, dark red curves) and after maximally six trials per target for the two-step sequences Fig. 4d). This is a novel observation where, different from the study by Gallego et al.^14^, here we have focused on the speed of re-calibration, which is a relevant parameter for any functional BMI. Two controls are also considered: As expected, one gets high performance when using the previous latent decoder on previous day latent space data (Fig. 4c–e, light blue curves). However, when applying that same pre-trained decoder directly to the unaligned latent trajectories from the current day, we found only chance level decoding accuracy (Fig. 4c–e, light pink curves). These findings emphasize the necessity to align the latent trajectories to be able to use pre-trained decoders.

A second substantial gain in computational efficiency, which facilitates the use of the decoder in embedded hardware, arises from the fact that matrix operations from the CCA transformations, the projection into the low-dimensional manifold and the weight matrix multiplication in the perceptron can all be combined. In particular, the perceptron input layer weight matrix of a pre-trained decoder (Fig. 4b, “Prev. Lat. Dec.”) can be adapted to also directly incorporate the CCA- and PCA transformations. A decoder adapted this way (Curr. Spike Dec in Fig. 4b) can, thus, be directly applied to the spiking dynamics from the second day while maintaining the same accuracy (Fig. 4c–e, light red). This holds both, for decoding single actions (Fig. 4c) as well as two-step action sequences (Fig. 4d), and even when both sequence types are combined into one data set and decoded together (Fig. 4e).

We conclude that the alignment of latent trajectories of brain activity provides an adaptation mechanism for a pre-trained decoder, which yields good decoding accuracy much faster than training a decoder from scratch. This, in turn, provides a key technology to render online BMI decoders usable, because only few calibration data are needed day by day. In addition to this, the fact that all matrix operations can be combined creates a very compact decoder—a small, augmented perceptron—which is compatible with edge device technology and low-power operation working directly on spike trains. Consequently, in the next sections, we will demonstrate that it is indeed possible to apply this method in real-time to a live experiment and that these methods are suitable for BMI applications.

### Modular software framework and embedded hardware implementation for online operation

**Figure 5:**
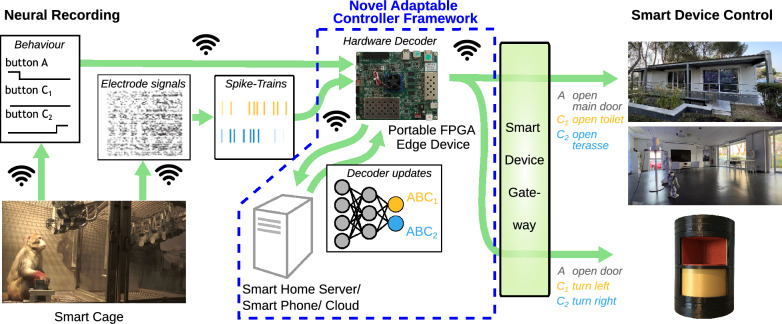
Overview of the experimental framework used for demonstrating proactive control: freely moving monkeys, which are implanted with wirelessly transmitting multi-electrode arrays to record their brain activity, perform a sequence of actions (wait-walk-reach) within a smart-cage, which also tracks their behaviour. Spiking activities are transmitted to a control framework comprising an edge device (FPGA) and a cloud server. The FPGA decodes the planned action sequence and issues commands via a middle layer that provides compatibility with multiple smart environments ranging from individual smart devices like a light or a smart cabinet (bottom right) up to the complete infrastructure of a smart home (top right). The cloud server uses spike-trains and behavioural signals from the smart cage as ground truth to perform the computationally heavy online adaptation of the decoder and then uploads the adapted decoder to the edge device. The behavioural signals provide a baseline for the sequence execution time, which can be used to quantify the proactive gain.

Using the above derived decoder and adaptation mechanism, we developed a modular software framework for performing proactive, online BMI control^23^. In contrast to the previous offline analyses of recorded data, the decoder inference and adaptation as well as the issuing of smart device commands are now performed online—that is using neural recordings that are performed at the same time and streamed via network. Yet, the controller architecture remains “open-loop”, as the animals do not perceive the actions of the smart-devices (see Fig. 5). Following an edge-computing design, we implemented a fast, feed-forward decoder module including an accelerated, energy efficient hardware implementation of the decoder being at “the edge” (Fig. 2, top, blue, ② and ③) and a cloud-based decoder adaptation module (Fig. 2, bottom, red, ④ to ⑦) with suitable interfaces between these modules.

**Hardware implementation of decoder module** Our decoder (Fig. 2, ③) works directly on incoming activity data from the brain collected together with behavioral data (Fig. 2, ②). The implementation of the decoder on hardware is using a Xilinx ZCU104+ FPGA, for which we have developed a FPGA driver that handles the input of feature vectors, the readout of the decoder results, as well as the deployment of decoder updates. We have evaluated that our newly developed FPGA implementation supports real-time decoding with over $30\,000$ decodes per second with a process-latency of maximally $32\,\upmu \text {s}$ and a power consumption of $246\,\text {mW}$. Thus, the system’s latency is negligible compared to the delays in the millisecond range for the wireless transmission of smart house commands. In addition, the dynamic power consumption of the FPGA implementation is orders of magnitude lower than the power uptake of any regular computer, which would consume up to 250–1000 times more energy^24^, or even of a Raspberry Pi (1st gen.) that consumes 20 times more energy. With a typical smartphone battery ($4000\,\text {mAh}$), our system would enable $30\,000$ decoding operations per second for $60\,\text {h}$. To evaluate further optimization potential for the energy consumption of the presented type of decoder, we compared our hardware implementation of an adaptable decoder with an optimized implementation of a non-adaptable decoder for an FPGA. We found that the non-adaptable decoder exhibits an energy uptake which is reduced by a factor 2–3 only^25^, such that further optimisation of the FPGA implementation is not expected to yield significant gains.

**Figure 6:**
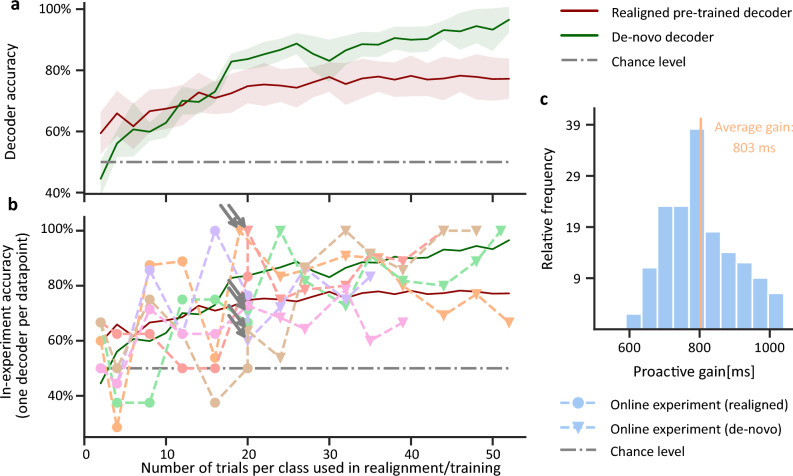
Accuray of online decoding achieved and potential proactive time gain achievable in our online, open-loop proactive BMI framework (**a**) accuracy of the pre-trained, adapted and the de-novo decoder implemented in our online BMI framework for different numbers of “calibration” trials per class (analogue to Fig. 3). The de-novo decoder requires data from more trials ($>15$) to achieve good decoding results whereas the adapted decoder surpasses chance level already with two calibration trials per class. However, different from the offline analysis, the de-novo decoder outperforms the adapted decoder when many trials are available. (**b**) Online accuracy estimates from different runs of our online framework. Each line indicates one online experiment, with each data point representing the accuracy estimate for the decoder update employed at the respective number of calibration trials. Circles indicate use of adapted decoders, wheres triangles indicate results after switching (arrows) to de-novo decoders. We see that the experimental accuracy estimate develops as predicted from (**a**). Note that the accuracy of the decoders during life experiments was estimated on-the-fly only based on the samples acquired before the next decoder update, resulting in larger fluctuations of the accuracy estimate when compared to the offline analysis. (**c**) Distribution of the achievable proactive gain for all trials in one online experiment.

**Figure 7:**
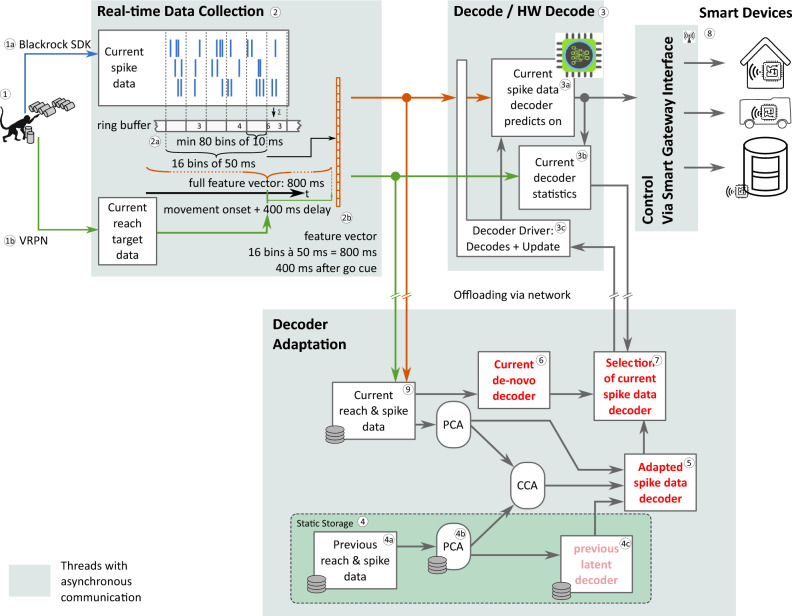
Online data flow for the software framework extending Fig. 5, focusing on differences to the offline-case due to the real-time data stream. I.e., the construction of feature vectors from the input stream, the updating of decoders in the running framework and the smart gateway interface. These details are described in the methods and supplement S2. Note: the numbering corresponds to that in Fig. 5.

**Decoder adaptation module** The decoder is updated by the decoder adaptation module. This module has two paths: in one path, the feature vectors are stored and used to update a decoder that is pre-trained by data from a previous session (Fig. 2, ④), by manifold realignment (Fig. 2, ⑤), while in the other path the feature vectors from the current session are used to directly train a de-novo decoder (Fig. 2, ⑥). Based on the performance of both decoder types, a selection mechanism (Fig. 2, ⑦) can switch between the de-novo and the adapted decoder (see Methods and Supplemantary Information S2). Due to the high computational demand, up to now, this module runs as software on a cloud server and sparsely links to the FPGA to receive new data and to send the updated decoder.

Taken together, we have developed a modular hardware-software framework, which is the first comprehensive integration of key-technologies required for exploring the advantages of proactive BMIs. Note that the individual components of our framework can be combined as needed, for example using an embedded edge-device paradigm, where decoder adaptation is performed by an assisting device such as a server or a smart phone or a single-device lab setup, where both processes are handled by the same device. Hence, this framework can be employed in experimental laboratory settings but also allows for future on-patient everyday use.

### Test setup for online decoding

Figure 5 shows the experimental setup for demonstrating online decoding functionality of the fully integrated hard- and software. We connected the neural recording equipment and the smart cage (Fig. 5, left) with the Xilinx ZCU104+ FPGA decoder by wireless transmission (Fig. 5, top, center). The decoding results of the FPGA were read out, translated into actions by a smart-device communication gateway^20^ and then wirelessly transmitted to the target smart devices (Fig. 5, right). Decoder updates (Fig. 5, bottom, center) commenced via the network as needed. With this setup, both communication with a local standalone smart device—a rotating smart cabinet (Fig. 5, bottom right)—as well as with a complete smart home via a long-distance communication (Living Lab Smart Home in Madrid^20^; Fig. 5, top right) were successfully tested.

The role of the communication gateway is to abstract over different smart environments, enabling easy transitioning from one environment to another. To this end, the gateway provides enumerations of devices and possible actions, enriched with semantic information, which allows to match and thus map action targets to smart devices based on ontologies. Furthermore, it allows feedback from the smart environment to the decoder, for example, to improve performance or to add new actions based on what the user is actually doing. These two features, the matching of decoded actions to smart device commands and the feedback from the smart environment, are key components for BMI control in complex human smart environments. As the gateway technology is built on open standards^26^, technical adoption is available to all interested parties.

### Online decoding demonstrates robust prediction on planning information

To test online decoding with sufficient statistics, we had the monkey perform the walk-and-reach action sequence to the two outermost targets. Decoding was performed $400\,\text {ms}$ after movement onset on feature vectors created on $800\,\text {ms}$ of neural activity binned over $50\,\text {ms}$ windows.

To analyse the online operation of our method, we quantify and compare the general performance of a de-novo decoder, which is trained from scratch on raw neural input, and an adapted decoder, obtained by manifold realignment. Already after two trials, the adapted decoder obtains an accuracy being significant better than chance level (Fig. 6a). As long as the number of trials stays small ($<15$), the adapted decoder consistently outperforms the de-novo decoder. We find that the hardware implementation of the decoder shows comparable performance to the offline analysis (Fig. 3f). After many trials ($>20$), however, the de-novo decoder provides a higher accuracy as the adapted decoder, initiating a switch in the adaptive decoder module to always use the most accurate decoder.

We next analyzed the online estimates of the current decoding accuracy obtained by our framework during individual runs (Fig. 6b), which are also used for the decision to switch from the adapted to the de-novo decoder. For each run, we show the accuracy and the decoder in use depending on the number of trials available for its training or realignment whereas the arrows indicate the time point of decoder switching. Note that these online accuracy estimates are only based on the few new feature vectors acquired between two subsequent decoder updates  (see Methods and Supplement S2). The size of these test sets is $\le 8$, as decoder adaptation is performed frequently to benefit from the high performance gain for small sample numbers. Thus, the online accuracy estimates are a coarse approximation and cannot be directly compared to the accuracy from offline analyses. However, the curves show that the also individual runs follow the same trend as our statistical analysis (Fig. 6a) and that our framework robustly employs the decoder with the best decoding accuracy.

In summary, we have demonstrated in online experiments that the proposed framework is robust and thus suitable for future BMI applications providing an accuracy that clearly exploits the advantage of adaptive realignment and dynamic decoder switching.

### Proactive BMIs result in faster action sequence control

Next, we tested whether the proactive BMI paradigm presented in this work would indeed allow a user to execute action sequences faster. For this, we assessed the proactive time gain—that is, how much faster a device-targeting command is issued as compared to waiting for the monkey to touch the respective target and trigger the command only reactively. We quantified this as the time difference between decoding of the reach target and the touch of the respective target and arrived at an average gain of $\approx 800\,\text {ms}$ (Fig. 6c). For experiments whose complete trials last $2567 \pm 256\,\text {ms}$, this is a significant reduction by $30{ \%}$. In this setting, the exact magnitude of the proactive gain is largely determined by the chosen time of decoding and the average sequence duration, here dominated by the time needed for the Macaque from taking up the initial position to walking towards the reach target.

Thus, we can clearly see a proactive gain in online experiments, which is significant when viewed in relation to the overall action sequence length, suggesting a substantial improvement for potential BMI users (see Figs. 1b, c and 6c, as well as the Discussion).

## Discussion

In this study, we have demonstrated the viability of a proactive BMI-control system in non-human primates and developed various key technologies with a human use-case in mind. In particular, we have presented a control architecture centered around a low-energy FPGA control device, performing the decoding of brain activities, which is integrated with (1) an action sequence setup for monkey experiments, (2) an online-adaptation mechanism for the decoder that is based on the alignment of latent neural trajectories and can be outsourced to powerful devices without energy constraints, as well as (3) a smart device interfacing gateway^20^ that allows the controller to work in various environments (Fig. 5).

Our adaptive decoder allows the framework to cope with the recording instability of intracranial electrode arrays which emerge from intermissions between multiple BMI sessions. In practice, such intermissions, which can stretch multiple days, also occur for decoding commands that are only used occasionally. In this case, tracking and correcting of incremental recording instabilities, as e.g. in^18,19^, is not possible and a quick recalibration as shown here is of central relevance for the usability of the BMI. Here we showed that the application of manifold realignment on very few calibration samples provides this rapid adaptation and results in a significant and continuous accuracy increase. This applicability of manifold realignment for fast calibration is also a new finding as it had so far only been applied on large prerecorded data sets^14^ or for continuous incremental adaptation of the BMI decoder^18,19^ and it can be assumed that such a fast calibration algorithm is relevant for any practical BMI use case relying on neural information suffering from inter-session variability.

While we present the integration of the fast calibration algorithm on weight level for the input layer of a multi-layer perceptron, it should be noted that the method is not tied to a specific decoder architecture. Integrating the transformation which results from the fast calibration algorithm is equivalent to adding one full-rank matrix multiplication at the beginning of the processing pipeline. When looking at artificial neural networks specifically, in all relevant frameworks, the integration can thus be achieved by adding an extra fully connected layer on the input side.

In contrast to offline experiments (Fig. 4), where realigned and de-novo decoders perform identically, the de-novo decoder achieves higher decoding performance with sufficient training data in our online framework. This is likely a combined effect of omitting the dimensionality reduction for the de-novo decoder together with the simpler online data treatment. At the same time, the training process of a de-novo decoder is orders of magnitude slower than the adaptation through CCA. Therefore our framework only switches to the de-novo decoder, when enough calibration data and training time are available. This method and framework for fast online calibration and switching in proactive BMIs is a central development of this study.

Based on these advances in decoding, our experiments demonstrate that proactive control is possible and can provide an advantage over other control schemes. To quantify this, we introduced the proactive gain as a measure of control efficiency. This measure, however, strongly depends on the considered control situation: (1) Given a larger smart environment or longer lasting actions within the sequences, the gain can be orders of magnitude higher than in our walk-and-reach example. To demonstrate the possible effect on a human scale, we show the difference between long lasting actions being executed in sequence or parallel for a human crossing two doors while switching on the lights along the way (Fig. 1a, Supplementary Video SV1) in the Living Lab, resulting in a proactive gain of around $8\,\text {s}$ or 38% (Fig. 1c, numbers taken from Supplementary Video SV1, toilet door case). Note that this gain is mostly governed by the movement of the actuators, which is chosen to be slow in an environment which is shared with humans. (2) As detailed for Fig. 3e, f, choosing an earlier time of decoding increases the achievable proactive gain, while it is lowering the average accuracy, resulting in a trade-off between proactive gain and decoder accuracy.

Nonetheless, the potential of proactive control goes beyond the faster execution of contextually linked action sequences. We see the proactive control paradigm as a game changer, as it allows to make the execution of abstract action sequences as unobtrusive as thinking about them, instead of orchestrating your smart environment through every step of a sequence explicitly. The consequence is that proactive control allows to make smart environments much more intuitive and interactive. By decoding sequences of actions, the proactive control paradigm implicitly approaches the targeting of an activity’s outcome, instead of the subject commanding and coordinating individual devices. At this point, this is merely a vision, which we expect to become a reality when real users are using BMIs in a closed-loop set up, where the decoding of planned actions might as well lead to a change in how the action sequences are planned, due to the feedback provided by the smart environment.

Our results also raise questions that have to be investigated before a possible human application. First, the experimental design of this study considered four possible actions with maximal one intermediate action (walking). However, it is unclear how the approach scales with more possible actions in a smart environment. On the one hand, more (diverse) actions might increase the dimensionality of the manifolds. Yet, as many motor behaviours seem to share the same manifold^27^, we are confident that this will not be a large problem. On the other hand, more possible actions could decrease classification accuracy and increase the amount of calibration trials needed. Here, it is crucial whether plans for multi-step sequences are encoded as a whole or whether the plan for each step of the sequence can be decoded separately (orthogonal encoding), as this determines whether required calibration trials scale exponentially or linearly with the number of actions and steps in the sequence. For instance for sequences of reach actions, the latter seems more likely^21^ such that the calibration effort may remain manageable.

Second, evidence for planning activity in the recorded areas mostly cover only one upcoming action (i.e. a one-step sequence) and it is unclear how many steps into the future can be decoded. Our data shows that the second-next action could be decoded, although the accuracy is lower until the first action has started. Thus, for long-ranging proactive control, additional implants in higher planning areas might be needed.

Third, the monkeys in our experiments reach for a touch target in the smart cage without knowing about the control of smart devices. Although the touch targets used in the experiment can serve as a model for electrical switches, buttons and even touch screens, which already represent a large part of the interactive elements in modern smart environments, it remains unclear whether the presented classification results will hold for interactions with real smart environments. Especially the duration of actions can be much longer, as illustrated in our time-gain example above. Yet, it needs to be tested whether the recorded areas hold planning information for such long time spans.

Finally, our decoding is open-loop as the monkeys do not receive feedback from the decoding. In a closed-loop system, such feedback can cause adaptation of brain activities and a different calibration behaviour of the decoder. On the one hand, the brain can then adapt to the decoder’s control outputs, which may speed up decoder training to help stabilise control^28–31^. On the other hand, the smart appliances could continuously supply signals of which actions have been triggered, providing the decoder with target information. Thus, a proactive controller could be calibrated while reactive control is active and deployed as soon as it gathered enough calibration trials. Therefore, the logical continuation of this work is to test our BMI framework in a closed loop environment with actual smart appliances before moving towards human application.

For the latter, one would furthermore have to devise methods to integrate user feedback and preferences into the control. Most prominently, users would need to be able to adjust the trade-off between time-gain and accuracy, but also to select actions for which proactive execution is wanted or unwanted. The latter would also be necessary for situations when a faithful control is critical such as in traffic. Also, as humans do not follow an experimental protocol, the time point of classification needs to be determined by the BMI. Here, smart environments can provide signals similar to the movement onset by broadcasting elicited commands. It is also possible to decode the intent and possibly plans from sensor data or action observations with cameras in the environment or on the user^32,33^. Finally, intent recognition can be achieved by decoding the neural activities themselves (extracranial^34^ as well as intracranial^35,36^).

To enable wide adoption of proactive control interfaces, smart home gateways are required to translate a sequence of actions to commands for multiple smart environments that a user encounters. As an example, a decoded action sequence for “making a cup of coffee” would always have to be matched and mapped to commands within the current smart environment if it provides the required facilities, although, e.g., the type of coffee machine may vary. These gateways also play an essential role in providing feedback to the BMI, which allows to adjust and extend its operation. We however did not integrate feedback from the smart home gateway, as our split approach with the monkey performing the task and the gateway executing the decoded and mapped actions in a human environment did not allow to make this connection. Research on closed loop BMI applications are therefore an important field for future research.

On the technical side, the next step towards mobile BMIs would tie the decoder directly to the recording equipment and use FPGAs and digital signal processors to implement improved read out, amplification, and post-processing of the neural data in combination with accelerated decoding. This would not only provide enormous potential for size and power reductions but also for improved online data processing in hardware, and thus better decoding. Interestingly, the fact that our decoder works on threshold-crossing events without spike-sorting entails that it might work with low resolution recordings or even event-based, removing the need for high frequency analog-to-digital conversions (ADCs) and high bandwidth data transfers. This provides potential for even more power saving (for the power consumption of an analogue front-end see^37^, and for the power consumption in a current integrated solution with $\approx 6~\text {mW}$^38^, both solutions still using high-frequency sampling and ADCs). Such a small integrated solution is also increasingly important as recording devices currently evolve towards larger channel numbers and higher electrode densities^38,39^. We therefore believe that our framework could contribute to maximally exploit the capabilities of these new devices.

In conclusion, the here-presented work provides important analyses and key technologies that show the viability and advantages of a proactive brain machine interface for mobile and multi-purpose human usage. These technologies have the potential to lead towards mobile, miniature assisting devices, which will not effect their users in terms of size and visual impact more than a hearing aid.

## Methods

This section is structured such that we follow the presentation of results addressing methods for (1) action sequence decoding via recorded neural activity, (2) fast online adaptation for re-calibration, (3) implementation of the framework for online BMIs with verification in online experiments. At the end, we shortly describe (4) the methods and tools used to create the embedded implementation of the adaptable decoder.

Note that while some methods apply to both, the offline and the online case, there are also methodological differences concerning data recording and processing, which are correspondingly marked as either off- or online and the differences are described. This two-fold description is necessary, because of two reasons. First, the online experiments can only verify the presented analysis and methods, but require offline analysis for thorough method development and validation with sufficient statistical analysis, which can not be performed with reasonable effort in the online case. Second, the online implementation targets simpler hardware specifications, which results in some simplifications.

### Neural recordings and behavioral experiments

#### Task and cage design

A detailed description of the experimental procedures can be found in^11^. The technology developed for the experiment is described in^40^. To assess multi-step action sequences, we use the walk-and-reach task-design, which had been developed to investigate planning of movements to targets, which are out of reach. It utilizes touch-sensitive LED lamps, two of which are used as start-buttons and mounted to the floor, four are mounted at the cage ceiling within immediate reach from the start buttons and another four are mounted at the ceiling at a distance, such that the monkey needs to walk towards them (Fig. 2, ①). Furthermore, there is a not-reachable lamp for an isoluminescent indication of the go-cue. All devices are controlled via an Arduino connected to a PC that runs the control software and handles network communication via the VRPN protocol. For offline analyses, all VRPN events and times stamps are recorded.

To engage with the task, trained monkeys signal that they are ready to begin a trial by touching both floor mounted buttons. Then, one of eight target lamps lights up to indicate the reach target of the current trial. After a variable waiting period, the go-cue is lit up and the monkey starts to move and touches the indicated lamp. The movement onset can be detected by the release of the start-buttons and the reach by the touch of the target. As the four close target lamps are in immediate reach range of the monkey, the brain activity during the waiting period encodes the plan for a one-step action. The other four lamps are further away such that the monkey needs to walk before touching them and the neural signals encode the plan for two steps of the action sequence. Thus, this task design allows us to assess proactive control and decoding of action plans for multi-step sequences. Both monkeys performed all reach movements very consistently ($100{~\%}$) with the left hand^11^.

Neurophysiological recordings were conducted in two adult male rhesus macaques (Macaca mulatta, K and L). The animals were group-housed in facilities of the German Primate Centere in Goettingen, Germany, in accordance with all applicable German and European regulations. The facilities provide the animals with an enriched environment, including a multitude of toys and wooden structures and natural as well as artificial light, exceeding the size requirements of the European regulations and including access to outdoor space. All experimental procedures comply with the German Law and the European Directive 2010/63/EU and have been approved by regional authorities (Niedersächsisches Landesamt für Verbraucherschutz und Lebensmittelsicherheit, LAVES) under permit number 33.19-42502-04-18/2823. All experimental protocols are in accordance with all relevant aspects of the arrive guidelines.

Both animals participated in a previous neurophysiological experiment^11^ and data from both animals was used for offline analysis. As part of these previous studies, monkeys were implanted with six 32-channel (= total of 192 channels) floating microelectrode arrays (Microprobes for Life Sciences, Gaithersburg, Maryland) with custom electrode length in the primary motor cortex (M1), the dorsal premotor cortex (PMd), and the parietal reach region (PRR) of the right hemisphere. Array connectors as well as recording equipment were protected by custom-designed implants^11,41^. The surgery protocol is described in^11^. The online decoding experiments were conducted with monkey L.

#### Neural recordings and post-processing

The recording of neural activity is performed by the commercially available Blackrock CerePlex Exilis (Blackrock Neurotech, Salt Lake City, UT USA) recording system, recording raw signals with $30\text { kHz}$, combined with two Blackrock Exilis wireless transceivers. In this system, the voltages from the implanted electrode arrays are pre-amplified, read out by the head stage and then transmitted wirelessly such that the monkeys can freely move. The wireless receiver is connected to a data acquisition computer, which can operate in two different modes: For online decoding, the voltage traces are thresholded to obtain spike events, which are then broadcast to the local network via the Blackrock’s Cerelink protocol. The threshold is determined as $-4.5 \times$ the root mean square of the signal during a trial. For online experiments, a high pass filter (1st order Butterworth with $300\,\text {Hz}$ cutoff) was applied during recording. For offline analysis, voltage traces are stored in a raw data format and the spike times are extracted later using offline post-processing as adapted based on^42^ .

For this, a sliding window median (91 samples window length, $~3\,\text {ms}$) was subtracted from the recorded raw signals and then a zero-phase second order Butterworth filter (cutoff at $5000\,\text {Hz}$) as applied. Then, to remove common noise sources shared by all channels, principal component artifact cancellation was applied per electrode arrays. Additionally, all principal components with a coefficient grater than 0.36 were retained (see^42^ and references therein). Finally, spikes are detected by threshold crossing and stored in a hdf5-file, which we use for offline analysis.

### Time-dependent offline decoding accuracy

Our aim is the decoding of planned actions within an action sequence. By task design, there is only a finite number of possible actions, this can be mathematically described as a classification problem—that is a mapping between feature vectors obtained from the neural activities in a defined time-window within each trial to the actions that they encode.

#### Offline feature vector extraction

For our offline analysis, we are using data from^11^, consisting of (a) recordings of the neural activities as well as (b) recordings of events in the smart cage that capture the given cues as well as the monkey’s behaviour. Offline analysis benefits from the availability of all data at all times. To obtain the feature and target vectors for classification, the information from both sources (a) and (b) is combined. To this end, we first extract successful trials from the smart cage recordings and obtain their time points of movement onset. Then, we extract spike times from a window with duration $T_{window} = 800\,\text {ms}$ determined relative to the last time-point in that window, i.e., $t_{end}=400\,\text {ms}$ after movement onset if not stated otherwise. We subdivide the extraction window into $n_{bins}=16$ distinct time bins of $T_{bin} = 50\,\text {ms}$ duration and count the spikes within each bin. This is repeated for each trial ($n_{trials}$ times) and for each of the $n_{channels}$ electrode channels. Hence, we obtain a (3D) data tensor with size: $n_{trials} \times n_{bins} \times n_{channels}$. As the classification algorithms used do not have an explicit notion of time the latter two dimensions were treated as one, such that we get a data matrix with $n_{trials}$ rows and $n_{bins} \cdot n_{channels}$ columns that are mapped to $n_{targets}$ reach targets by our decoder. Hence, we treat the activity in the different time bins of one channel as equally independent and informative as the activities from different channels. This enables classifiers without a notion of time to discern not only cases where the activity in some of the channels differs from target to target but also cases where the time course of the neural activities is differently shaped.

For extracting the latent manifold via PCA, we concatenated all trials such that we obtain a data matrix with $n_{trials} \cdot n_{bins}$ rows and $n_{channels}$ columns.

#### Time dependent decoding accuracy from offline neural data without spike-sorting

As different classifiers make different assumptions about the data and, thus, achieve different decoding performance, we decided to train a variety of classifiers and compare their results. While a much broader variety of algorithms and hyper-parameters was tested, we here show the results from the following classifiers:

- linear support vector machine (similar to that used in^11^),
- 3-nearest neighbor classifier,
- classifier based on linear discriminant analysis
- naïve Bayesian classifier, and
- multi-layer perceptron with one hidden layer comprising 50 neurons.
- multi-layer perceptron with two hidden layers comprising 50 neurons, each.
- multi-layer perceptron with one hidden layer comprising 100 neurons.

For all classifiers, standard implementations of the scikit-learn-package^43^ (version 1.3.0) were used. The classifiers were trained on $80{ \%}$ of the feature vectors and their accuracy was evaluated on the unseen $20{ \%}$. This was repeated 20 times using a k-fold stratified cross-validation strategy to obtain means and standard deviations for the classification accuracy.

To discriminate between areas, this training process was repeated for feature vectors that only contained the activities from electrodes in specific brain areas (M1, PMd, PRR or all together).

To assess the temporal evolution of the planning information throughout a trial, the whole process was repeated for feature vectors extracted with a broad variety of time shifts with respect to the movement onset (latest time in window varying from $t_{end}=-700\,\text {ms}$ before to $t_{end}=1000\,\text {ms}$ after movement onset).

### Offline test for decoder adaptation via manifold alignment

To test whether using a pre-trained decoder with manifold alignment is faster than training a new de-novo decoder, we first conducted an offline analysis. For this, we used two pre-recorded data sets, one of which is used as the “current day data set” and the other as a “previous day data set”. Assuming that only a limited number of trials have been recorded in a current session and are “available” for decoder calibration, we sampled a varying number of trials for each reach target from the “current” full data set.

In the following we describe the three main decoder variants which have been compared to each other (full implementation details of these decoders and the controls in the Supplementary Material). Again, decoder training and implementation used the scikit-learn framework (version 1.3.0).

We first trained a de-novo decoder (Fig. 4c, d, green curves; ①) on the data feature vectors from these sampled “available” trials and evaluated its prediction accuracy on the rest of the “current” data set. For statistical evaluation, the sampling and training was repeated 20 times. A flow diagram for the de-novo decoder is shown in Supplementary Information S1, Figure S1. Moreover, we also evaluated the accuracy of the de-novo decoder on the full current data set and depicted all other accuracies relative to this reference value (Fig. 4c–e). Note, while the de-novo decoder serves as a control for the offline analysis, it is also used for online decoding (Fig. 2) as soon as its performance supersedes the realigned decoder.

Second, to obtain a realigned decoder, we implemented the method for decoder generalisation through manifold realignment from^14^. An overview flow diagram for this is shown in Fig. 4a, more details are given in Supplementary Information S1, Figure S2. In short, we pre-trained a decoder on the latent trajectories of a previous day data set (left blue arrow to prev. lat. decoder, Fig. 4a). We then obtained a mapping from the neural activities to the current day latent space (lat. dyn. mappg.) as well as the current latent trajectories using the first 50 components of a principal component analysis (PCA) on the sampled trials. We then applied canonical correlation analysis (CCA) to obtain mappings that make the current and previous latent trajectories maximally correlated (CCA mapping). Note that both, the mapping to the current day latent space as well as the CCA transformations, are derived only based on the “available”, sampled trials from the current data set. For evaluation, however, we projected the full current day data set onto the previous day latent space. Then, we evaluated the prediction accuracy of the pre-trained decoder on these remapped latent trajectories (remapped curr. lat. traj.). Again the procedure was repeated for 20 different samplings of the “available” trials. Note, that only data is re-mapped and then fed to a previous day pre-trained decoder (upwards blue arrow to prev. lat. decoder, Fig. 4a) operating in the low-dimensional latent space ②.

Third, related to this, we used manifold alignment to adapt our decoder for direct use with spike data (Fig. 4b, see also Suppl. Inf. S1, Figure S3). For this, we multiplied the first layer weight matrix of a pre-trained decoder (on previous latent trajectories) with the CCA transformation matrices used to project from the current latent space to the previous latent space as well as the projection matrices from the spike rate data to the current latent space in the first layer. Hence, all data transformations are included in the final decoder and its prediction accuracy was evaluated directly on current day spike data (Fig. 4b, curr. spike dec., ③). Again the sampling of the trials used to obtain the transformations was repeated 20 times and the transformations were applied to the full current data set to obtain better statistics.

As two controls (Fig. 4c, d, light colored curves; ④ and ⑤), we also evaluated the prediction accuracy of the previous day data pre-trained decoder on previous day data (Suppl. Inf. S1, Figure S4) as well as on current day data, hence without manifold realignment (Suppl. Inf. S1, Figure S5).

### Adaptable controller implementation in the online software framework

The online software framework differs from the above offline analyses, due to its design for (a) real-time application and (b) the possible use in embedded hardware. We shortly summarise the differences here and refer the interested reader to Supplementary Information S2, the Supplementary Video SV2, and the publicly available code^23^ for more detailed descriptions and reference.

#### Online data collection and feature vector generation

Real-time data collection (② in Figs. 2, 7) combines information from the neural spike recording  with information about the smart-cage environment . Incoming spike data is continuously rate coded on $10\,\text {ms}$ blocks and collected in a ring buffer . We selected the “movement-onset” signal from the reach cage to trigger the creation of a feature vector with the same structure as in the offline analysis, , which is then passed on for decoding. I.e., each $50\,\text {ms}$ bin is constructed from 5 rate coded $10\,\text {ms}$ blocks by summation over the channels. Data collection and feature vector creation are performed in a timed low-latency loop to ensure real-time processing of incoming data.

As the timing of the data collection loop is crucial for the quality and the timing of the resulting feature vectors, no other processing is performed in this loop to avoid unpredictable delays. Instead, all other processing is performed in separate threads which are communicating asynchronously via message queues. Hence, the real-time data collection module sends the created feature vectors, together with the collected behavioural data (e.g. the selected target) to separate threads. Due to this separation, the other threads can perform tasks with variable delays.

#### Online decoding and smart house gateway interfacing

One of these separate threads is the decoding module (③ in Figs. 2, 7), comprising a realisation of the adaptable controller  and handling the mapping of the decoding results to trigger an action to be executed by the smart house gateway (⑧ in Figs. 2, 7)^20^.

As described above, a pre-trained decoder based on a multi-layer perceptron was selected for this study, using one hidden layer with 20 neurons. This decoder predicts full action sequences and controls the smart house or a smart cabinet, respectively (Fig. 5 and Supplement S2, where hyper-parameter selection and the differences between offline analysis and online trials are detailed).

For our experiments, the mapping from monkey actions (① in Figs. 2, 7) to a human-centred smart house or smart cabinet (⑧ in Figs. 2, 7) had to be pre-determined and, thus, the corresponding control commands were looked up based on the decoded reach target of the monkey.

After decoding the planned action sequence and mapping to the human-centred environment, the smart home gateway interface^20^ is used to initiate actions using a hypertext based json rpc interface over TCP/IP. The used technologies are all standardised and include a standard wifi network card for device interconnect and a curl based software library for issuing actions, as well as standardised protocols for transport and device enumeration. The curl library establishes a hypertext transport protocol connection (plain http or secured https) to either the smart home gateway, or directly to a local device. As described in^20^, the smart home gateway provides descriptions of controllable entities using the Web of Things Thing Description standard version 1. From these descriptions, the URL and the json content for the http put request can be constructed.

In^20^, the latency of the secured smart home gateway interface was established as $\approx 10~\text {ms}$ by determining the mean round-trip time from issuing the action until status change for a self developed device, with and without the gateway. This latency is comparable to local network round trip times as measured via ping, which will further depend on the network coverage and quality. The mean latency for the execution of a whole device command depends very much on the device. For a smart cabinet developed during the project, the command execution time was determined to be $\approx 243~\text {ms}$ with and $\approx 234~\text {ms}$ without the gateway infrastructure. This latency can be considered short in relation to the execution time of complex human-environment actions taking seconds and the operation of larger actuated devices in a direct human-machine interaction setting, which are typically actuated slowly to reduce the risk of injury.

### Decoder adaptation module

The decoder adaptation module handles both the time and memory consuming processes of calibrating pre-trained ( in Fig. 7 to ⑤ in Figs. 2, 7) and creating de-novo decoders (⑥ in Figs. 2, 7). For calibration, a static storage ④ provides previous reach & spike data, bundled with the PCA transformation and the previous latent decoder. These are used together with the newly acquired data ⑨ to perform the CCA transformations and manifold realignment. New de-novo decoders are trained in parallel when sufficient current reach & spike data was accumulated to allow the formation of a validation set to estimate the de-novo decoder’s performance. The first de-novo decoder is created for 20 current feature vectors per target, based on the condition that we need at least two elements per target for validation when using $10{ \%}$ of the feature vectors as validation set. A comparison between the de-novo decoder’s validation performance and the estimated adapted decoder’s performance determines which decoder will be used to update (Fig. 7, ) the current spike data decoder (Fig. 7, ) (described in detail in Supplement S2). Due to these processes being time consuming and of variable run-time, the decoder adaptation module is designed for offloading to another machine to increase the battery run time in mobile devices which will also result in a smaller form factor in mobile applications.

Decoder adaptation and training have been implemented in accordance with offline methods. Instead of scikit-learn, we used armadillo (versions 9.400 - 12), ensmallen (versions 1.10-2.20), and mlpack (version 3, git development revisions due to concurrency issues which were only published in mlpack version 4). Manifold-alignment has been tested to be numerically indistinguishable for the off- and online cases. For creating pre-trained decoders, the ADAM optimiser and its parameters were matched to the parameters from the scikit-learn implementation used in the offline analysis. More details on hyper-parameter selection and the differences between the offline analysis and online trials can be found in Supplement S2.

### Online Decoding Experiments

To validate the performance of the presented method in an online setting, in comparison to the statistical offline analysis presented in the results section, we stored the feature vectors together with the target labels and recorded accuracy estimates of the current decoder with the proactive gain during an experiment. All trials with monkey actions deviating from the instructed target were excluded from the decoder statistics due to the ambiguity in the neural recording.

The proactive gain was measured as the time difference between the moment of successfully decoding the target action and the monkey touching the correct target. Therefore, trials with inaccurate prediction by the decoder were not considered for determining the proactive gain.

Note that in the online experiment, we estimate the accuracy of the adapted decoder on feature vectors not used during the realignment or training. Hence,due to the high update frequency of the calibrated pre-trained decoder, typically only up to 8 newly generated feature vectors are used to estimate the accuracy before the next realigned decoder is employed. This leads to a coarse online estimation of the accuracy with typical values of ${^{n}/_{8},\,n \in [1, 8]}$.

To overcome this and perform a rigorous evaluation procedure for our online experiments, we generate statistics on stored feature vectors acquired by our system over the $\approx 2\,\text {h}$ experiment period analogue as in our offline analysis. This way, realignment with the online decoder implementation on stored online feature vectors could be repeated 20 times for different numbers of feature vectors, providing similar statistics as in the offline case. To this end, the whole exported current day data set with 58 feature vectors per target was split into an alignment set with the desired number of feature vectors per reach target *n* and the remainder was used to test and evaluate the accuracy of the realigned decoder, analogously as for the offline analyses. To also include variations stemming from the pre-trained decoder, for each of the 20 repetitions and for each value of *n*, also a new “pre-trained decoder” was trained (on the same previous day data set that had been used for the online experiment) .

In addition, a new de-novo decoder was trained on a training set with the same number *n* of feature vectors used to align the latent trajectories. Also here, feature vector selection was completely randomised and repeated for each of the 20 repetitions.

Note, different from the offline analyses, all these decoders were trained and evaluated within our software framework and thus correspond to the actual algorithms applied in the online experiments.

Online decoding was performed with different hardware bases. 1) The main software-only tests were performed with a powerful, dedicated computer in the local network, connected via ethernet gigabit ethernet. The machine is equipped with $128~\text {GB}$ of RAM and an AMD Ryzen ThreadRipper featuring $48~\text {CPU threads}$ and local transmission latency was estimated via an ICMP ping to be in the range of $<5~\text {ms}$. 2) The same machine was used as cloud end-point for the egdge setup, operating online and transferring the re-trained and de-novo decoders to the FPGA development board over the network. The decoder transfer was implemented using an industry standard TCP ssh file transfer with public key authentication. 3) Further software tests were conducted with a Raspberry Pi v3, which was still capable of performing the task, despite the training of de-novo decoders being completed on very long time scales.

### Accelerated hardware decoder implementation

Accelerating machine learning methods with hardware implementations comes into play when targeting applications for small form-factor, low power mobile devices. Whereas method development is typically performed on powerful machines, often with high end graphics processors, mobile applications live from batteries and require energy- and form-efficient solutions. When targeting, e.g., mobile medical applications or BMIs, residual heat can be another concern.

To address this, an FPGA chip was selected for the hardware implementation of the presented decoder ( in Figs. 2, 7). It saves energy and space being also heat efficient, while still allowing to rewire the hardware implementation easily. Furthermore, circuits developed with FPGAs can be mass-produced as even more efficient ASICs, if desired at a later point.

We obtained our optimised hardware decoder implementation  for calibrated pre-trained decoders using a tool specialized for adaptable multi-layer perceptrons, which was developed for this purpose^25^. This hardware decoder is integrated by means of a hardware driver , which orchestrates 1) the decoding of feature vectors as created by the real-time software stack (Fig. 2, ②), as well as 2) decoder updates by means of updated weight matrices, as created by the asynchronous decoder adaptation module (Fig. 2, ⑦).

Along this line, successful decoder adaptation by manifold realignment only updates the hardware decoders’ input layer weight matrices to include the transformation matrices gained via PCA and CCA (as described in eq.(1) in Supplement S1), whereas a new de-novo decoder requires a full update of all weight matrices.

The hardware implementation, thus, complements the software framework and enables immediate application on mobile, embedded hardware.

## Supplementary Information

Supplementary Information 1.

Supplementary Information 2.

Supplementary Information 3.

## Data Availability

The datasets used and/or analysed during the current study are available from the corresponding author on reasonable request.

## Code availability

The C++ code for the online decoding & adaptation framework will be made available at https://github.com/plan4act^23^ upon publication.
