# Supplementary material for: A brain machine interface framework for exploring proactive control of smart environments
Source: Sci Rep. 2024 May 14;14:11054. doi: 10.1038/s41598-024-60280-7 (PMC11584623; doi:10.1038/s41598-024-60280-7)
Supplement: Supplementary file 1 — Supplementary Information 1. [file 41598_2024_60280_MOESM1_ESM.pdf]

Supplementary Materials for

Proactive Brain Machine Interface for the Control of Smart Environments

Jan-Matthias Braun , Michael Fauth, *et al.*
*Corresponding author Email: j-mbmmmi.sdu.dk

This PDF file includes

Supplementary Texts S1 & S2
Figures S1 to S7

Other Supplementary Materials for this manuscript

Videos SV1 to SV2

Supplementary Texts

S1 - Protocols for classifier generalisation

In the following we describe the protocols and data transformations that were conducted to compare training a classifier from scratch and the use of manifold realignment to utilize pre-trained classifiers

The de-novo decoder

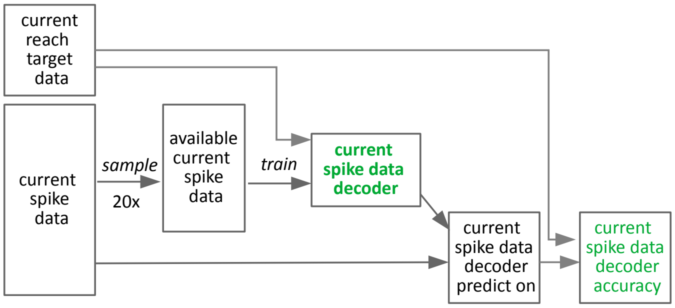

Figure S1. Basic Control Setup: Structure and process to evaluate a de-novo decoder

The de-novo classifier (Figure S1) serves as the basic control for all other decoders. For this, we use a multi-layer perceptron, which is trained and evaluated in the following way: We sample a certain number of training trials from the complete current day data set (80%) and then train the decoder (current spike data decoder). We then evaluate its accuracy in predicting the unseen part of the current day data set. To generate statistics, we repeat this process 20 times, each time using a different sampling.

General methods required for decoder generalisation through manifold realignment

We compare the de-novo decoder with various variants of methods, which are all based on manifold realignment[1]. For these methods, we always have to 1) find the low dimensional latent manifolds, 2) project the data onto them to obtain the latent trajectories and 3) find projections that align the latent trajectories. In the following we describe these steps using the diagram in Figure S2 showing the first type of aligned decoders, which has been adapted from[1].

1) Obtaining the low-dimensional manifolds: As a first step, we determine the low dimensional manifolds by which most of the variance of the neural activity can be explained. We assume that these manifolds are linear (i.e. low-dimensional hyper-planes in neural activity space). In this case, the manifolds explaining most of the variance can be found using principal component analysis (PCA). To apply PCA, we use the methods for data preparation described in the main text and concatenate the neural activity data from all time bins in all considered trials. Thus, we perform a PCA on

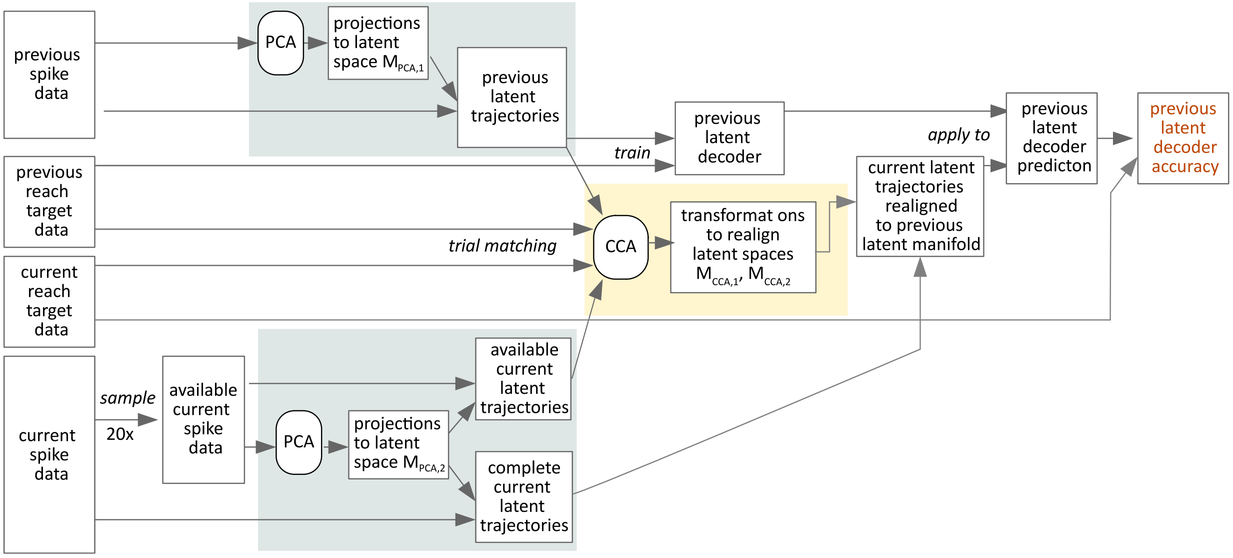

Figure S2. Aligned Decoder Type 1: Process to evaluate decoder generalisation from a previous day following[1]. First latent manifolds and trajectories are determined (grey boxes). Second, realignment transformations between the latent trajectories are extracted via CCA (yellow). Third these transformations are applied to the current day latent trajectories to transform them into the previous day latent space and apply a decoder trained on previous day latent trajectories.

$n_{trials} \cdot n_{bins}$ data points from an $n_{channel}$-dimensional space. We determine the first n_{PCA} principal components that span our manifold; hence, the projection matrix $M_{PCA,i}$ that projects the neural activities to this manifold (Figure S2, grey box center). Here $i \in \{1,2\}$ stands for the projection matrices to the latent spaces for the previous and current day data sets respectively. For the current day data set, we assume that only a sub-sample of the trials are available. Thus, we sample a given number of trials for each reach target from the current day data set (Figure S2, bottom left). On those the transformations are calculated (Figure S2, bottom center). We commonly used $n_{PCA} = 50$ components, which cover more than 80% of the variance, but we also verified that the results do not significantly improve, when more dimensions are taken into account (data not shown).

2) Obtaining latent trajectories: We then use the PCA-projection-matrix on the neural activity data tensors to obtain the projections onto the low dimensional latent manifold, which we refer to as the latent trajectories (3D-tensor with $n_{trials} \cdot n_{bins} \cdot n_{PCA}$ dimensions). This process is repeated for both data sets (Figure S2, grey boxes). For the current data set, we obtain the projections both, for the "available trials" as well as for all trials ("all data") of the recording session. Note, however, that the transformations in both cases are based on the limited sub-sample of "available" trials (Figure S2, bottom grey box).

3) Realigning manifolds: In principle, the first n_{PCA} principal components should identify the same low dimensional manifold of the neural activities in each recording of the same monkey. However, the neurons from different recording session provide different "views" onto this manifold, such the projected traces may be stretched, mirrored or rotated versions of each other. Thus, we next determine linear transformations that compensate for this. For this, we select trials corresponding to the same action sequence in both recording sessions ("trial matching") and concatenate the PCA-projections of the neural data from these trials, thus, obtaining two trial-matched latent trajectories data sets. We then use canonical correlation analysis to determine linear transformations $M_{CCA,1}$ and $M_{CCA,2}$ which map from each of the n_{PCA} dimensional latent manifolds into a common CCA space, such that the projected data in this space become maximally correlated (Figure S2, yellow box). By construction, both PCA spaces have the same dimension and the linear transformations are $n_{PCA} \times n_{PCA}$ matrices that can be inverted. Hence, the CCA transformations essentially provide a direct mapping between the latent manifolds (PCA spaces) from both data sets.

Aligned Decoders

The above described steps are common to all of the aligned decoders, which will be described next.

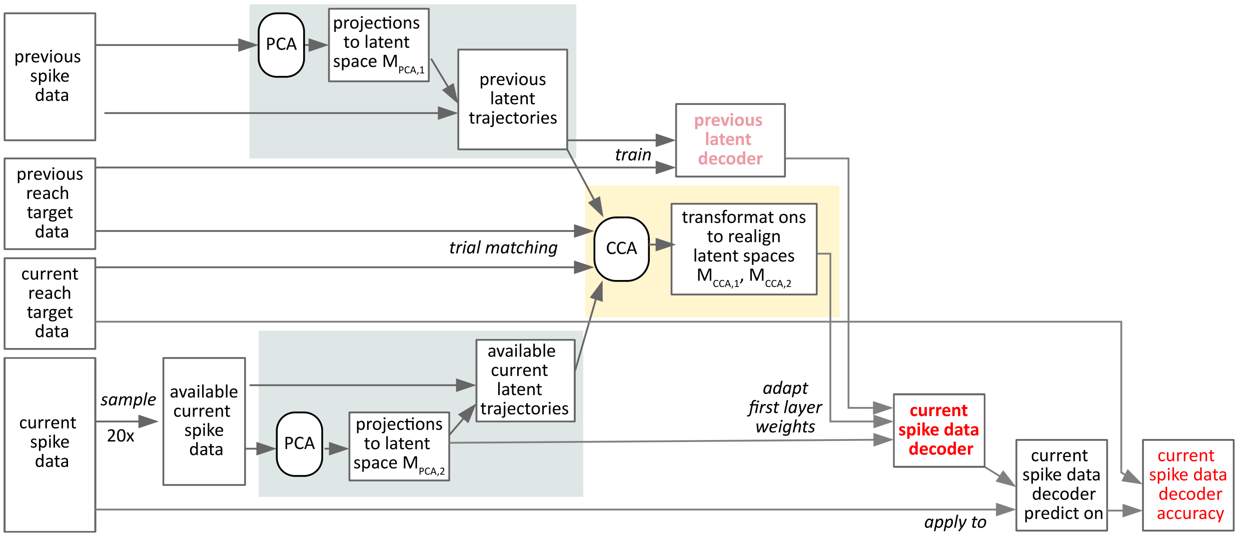

Figure S3. Aligned decoder Type 2: Process to adapt a pre-trained decoder via manifold realignment. First latent manifolds and trajectories are determined (grey boxes). Second, realignment transformations between the latent trajectories are extracted via CCA (yellow). Third these transformations are used to adapt the first layer weight matrix of a decoder trained in previous day latent space. The resulting decoder can directly be applied to the current day spike data.

Aligned Decoder Type 1: Pre-trained decoder employed on transformed data[1]

The first type of advanced decoders (Figure S2) follows first the above described steps. From there on, following the method proposed in[1], the current day PCA-projections (latent trajectories) are projected to the PCA space from the previous day using $M_{CCA,1}^{-1} \cdot M_{CCA,2}$ (Figure S2, right). We then trained a classifier on the previous day latent trajectories (Figure S2, previous latent decoder) and applied it to the so transformed current day data. Hence, the classifier is trained without any knowledge of current day data.

Note furthermore that in[1] the transformations into the current day latent manifold and the realignment were based on the complete current day data set. Here, although we evaluate the accuracy of the classifier on the complete transformed current day data set, both PCA and CCA transformations are only based on the "available" trials (Figure S2, bottom right), thus, also evaluating the "future" decoding performance if no more changes were made to those transformations. Again, for each number of available trials, 20 samplings and the respective transformations and decoder training were performed to obtain statistics.

Aligned Decoder Type 2: Pre-trained decoder adapted to be employed directly on spike data

We also tested whether manifold alignment can be applied to adapt pre-trained classifiers in order to directly apply them to currently acquired spike data. Again, we determined the low dimensional manifold of the current day data as well as the CCA transformation matrices $M_{CCA,1}$ and $M_{CCA,2}$ based only on a restricted set of trials available from the current day. To ensure the same number of samples, we bootstrapped the limited data available from the current day. Processes up to this point (yellow box in Figure S3) are identical to those in Figure S2:

Again, we pre-trained a classifier on the previous day latent trajectories. However, different than for the aligned decoder type I, we included the CCA transformations as well as the projection of the current day data to its low dimensional manifold into the first layer weight matrix W_1 of that decoder such that it can be applied directly on the neural activity feature vectors from the current day:

$$W_{1,adapted} = W_1 \cdot \mathscr{B}_{n_{bins}} \left(M_{CCA,1}^{-1} \cdot M_{CCA,2} \cdot M_{PCA,2} \right) \tag{1}$$

Hereby, $\mathscr{B}_{n_{bins}}(X)$ is a block diagonal matrix consisting of n_{bins} repetitions of matrix X along the main diagonal. This becomes necessary as our feature vectors comprise multiple time-bins and data from each of the time bins needs to undergo these transformations individually. Thus, we have arrived at the aligned decoder Type II (Figure S3 "current spike data decoder", bold red). While the results are mathematically identical to transforming the data, this procedure provides a way to use manifold realignment to adapt a pre-trained classifier for direct use with current day spike data, which makes it better suited for embedded implementation.

Also for this decoder, evaluations of the accuracy have been done on the whole current data set and repeated for 20 samplings of the available trials from current day to obtain sufficient statistics.

Controls

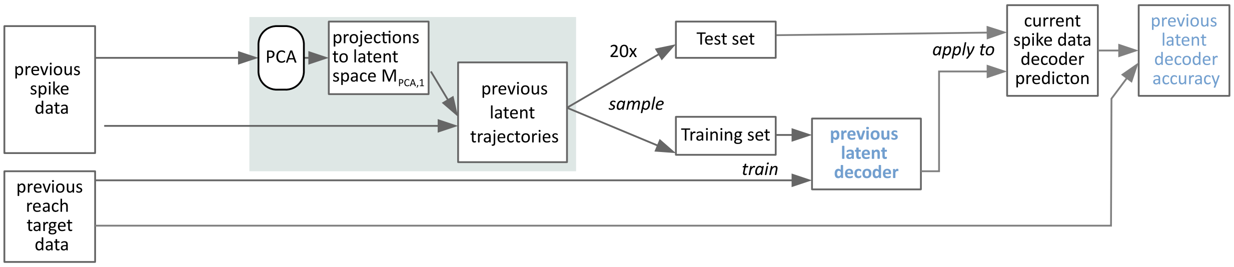

Figure S4. Control I: Process to test the maximal accuracy on a pre-trained decoder trained and evaluated on previous day latent trajectories.

Control I: Pre-trained (previous day latent) decoder on previous day data

As a first control, we evaluated the accuracy that the pre-trained classifier could reach on the previous day latent manifold projections. To this end, we first determined and executed the projections to the previous day latent manifold (Figure S4). We then trained classifiers on 80% of these previous latent trajectories and evaluated them on the remaining 20% (Figure S4). This splitting and training was repeated 20 times using a stratified 20-fold cross-validation scheme.

Control II: Pre-trained (previous day latent) decoder on unaligned current day latent trajectories

As a second control, we determined whether realignment was indeed necessary. For this we first determined the latent trajectories and manifold projections for the previous data set and a the available current day trials (Figure S5 grey boxes), but did not determine the realignment transformations via CCA. Instead we evaluated the accuracy of a classifier trained on the previous day latent trajectories applied directly on the current day latent trajectories. Here again, the latent manifold of the current day was determined only based on a sub-sample of available trials, while the accuracy evaluation was done of the complete projected data set and the set of available trials was sampled 20 times for each number of available trials to generate statistics.

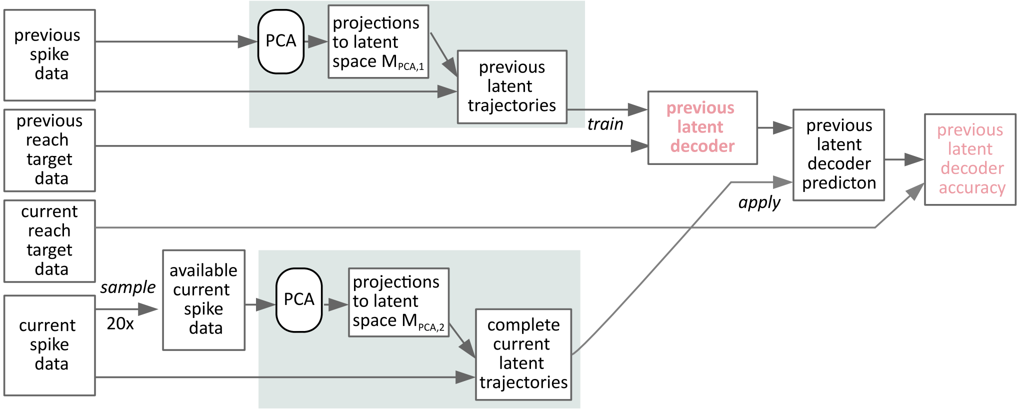

Figure S5. Control II: Process to determine classification accuracy without manifold realignment.

S2 - Detailed Description of the Software Framework Architecture

In this supplement, we describe the modular hardware-software framework in more detail, especially the choices made in addition to the general description in the methods section. We published the framework at[2], to make the presented methods accessible to the community in the hope that it helps to strengthen research in proactive BMIs.

For general application, we have designed the framework such that it is not bound to a specific setup, smart environment, or decoder architecture. As a consequence, the framework is set up in a modular way (Figure 7). All experiment-specific parts use generic interfaces and can easily be exchanged. The same is true for software decoders, for which many toolboxes exist, e.g.,[3] which was used in this study. Integrated into the framework is an optimised hardware implementation of a multi-layer perceptron, which can also be replaced. Still, optimised hardware implementations are not available in the same variety as software decoders. Note however that similar automatised optimisation techniques have also been used to design hardware implementations of recurrent echo state and radial basis function networks[4–7], but have not been integrated into the framework yet.

Online data collection and feature vector generation

As described, the data collection for decoding is triggered by the "movement-onset" signal from the reach cage, which initiates a $t_{end} = 400$ ms delay after which the neural data of the previous 800 ms needs to be rate coded and binned into a feature vector that can be interpreted by the decoder. Thus, data collection needs to combine information from two independent information sources: 1) the environmental and behavioural information (Fig. 7, ⓵b), communicated by the smart cage via the VRPN protocol[8], and 2) the neural spike recording (Fig. 7,⓵a), built on top of the Blackrock CereLink SDK. As this information is communicated in network packages (using the UDP protocol), it can arrive slightly delayed. Thus, data collection would need to handle the synchronisation of the independent data sources' internal clocks as well as the matching of data with the same time-stamp from both data sources. However, during experiments the effective delays in local network communication were determined to be on the order of a few milliseconds. Based on the observation that shifting a feature vector by up to 50 ms has little effect on the resulting accuracy (see, e.g., Fig. 3 **e** and **f**), we decided to avoid clock synchronisation between the behavioural and neural data streams (Fig. 2, ①) and assumed that the incoming data streams are roughly synchronised, when employing low-latency i/o to achieve real-time communication with regards to these timing constraints. This assumption allows us to simply count the spikes transmitted in a time interval T_{loop} instead of keeping track of their timing, which would involve dynamic memory allocations, copying operations, as well transformation and comparison of the time stamped data. Through this counting of spikes in an interval, we also directly obtain rate coded data which we continuously store in a ring buffer of sufficient size (Fig. 7, ⓶a).

Importantly, the loop traversal time T_{loop} determines the data collection frequency $\nu = 1/T_{loop}$ at which our data collection loop looks for newly arrived spikes as well as environmental and behavioural information. Note that this data collection frequency has to be chosen sufficiently high, as it determines the time resolution of the data acquisition and, thus, also the alignment of the feature vector with the behavioral signals. Along this line, the size of each time-bin in the feature vectors T_{bin} is necessarily an integer multiple of the loop time: $T_{bin} = N \times T_{loop}$ with $N \in \mathbb{N}$ and so is the window length for generating the feature vector $T_{window} = n_{bins} \cdot T_{bin} = n_{bins} \times N \times T_{loop}$. On the other hand, if ν is chosen too high, the processing overhead from the interaction with the interface library provided by the manufacturer of the recording hardware will dominate time spent on data collection.

Considering this trade-off, we chose $T_{loop} = 10$ ms . Hence, with $T_{window} = 800$ ms and $n_{bins} = 16$, we obtain $T_{bin} = 50$ ms and one value of the feature vector is created by summing up $N = 5$ buffered spike counts. To tune the timing of our i/o-loop, we measure the time elapsed while traversing the loop to tune the loop delay, through which the target loop time can be achieved. As the timing of the data collection loop is crucial for the quality and the timing of the resulting feature vectors, run-time measurement of the data collection loop traversal time ensures that the delay between successive data fetches is correctly timed. Moreover, all further processing is performed in separate threads. These are the decoding module (Fig. 7,③) and the decoder adaptation module (Fig. 7,④-⑨), which receive the created feature vectors, together with the collected behavioural data, when available.

Online decoding & smart house gateway interfacing

Online decoding, in combination with a separate driver for the decoder hardware, handles the output of the BMI. A message passing architecture ensures non-overlapping actions, i.e., decoding and decoder updates. In case of a software decoder, this process is trivial. For a hardware accelerated decoder, the decoding module (Fig. 7,③) communicates directly with the decoder driver and hands over feature vectors and firmware updates, while receiving decoding results. The hardware decoder supports two independent memory regions for decoder weight matrices, which can be switched on-the-fly after the comparably slow process of rewriting the weight matrices in FPGA memory, to ensure consistent decoder weights during the process.

Decoder adaptation module

Due to the separation of the real-time BMI control and the decoder adaptation code paths, especially in the edge device setup over the network, the time consuming adaptation process can run unconstrained, communicating updated decoders when ready. The critical issue is the time of switching from adapted to de-novo decoders due to the lack of a suitable test set to compare both decoders.

The adapted decoders generally use all available feature vectors for calibration—it is this key feature which allows fast adaptation. In consequence, the last adapted decoder's performance is used as a proxy for determining the adapted decoder's accuracy. Regarding the de-novo decoder, we determine the earliest time of switching to be at ≈ 20 feature vectors per target by the requirement that, with a $9:1$ training:validation split, at least 2 feature vectors per target are available for validation, which is still very low and will initially provide rough estimates which are subject to larger fluctuations due to the bad statistics during validation.

In practise, two ways of operation have been tested. First, we tested a sequence of decoders, requiring the de-novo decoder to be superior to the adapted for at least 3 successive trials. In practice, this approach suffers from the bad estimation of the decoder accuracy due to the small validation set which is stabilised to some degree by the requirement on the minimum size

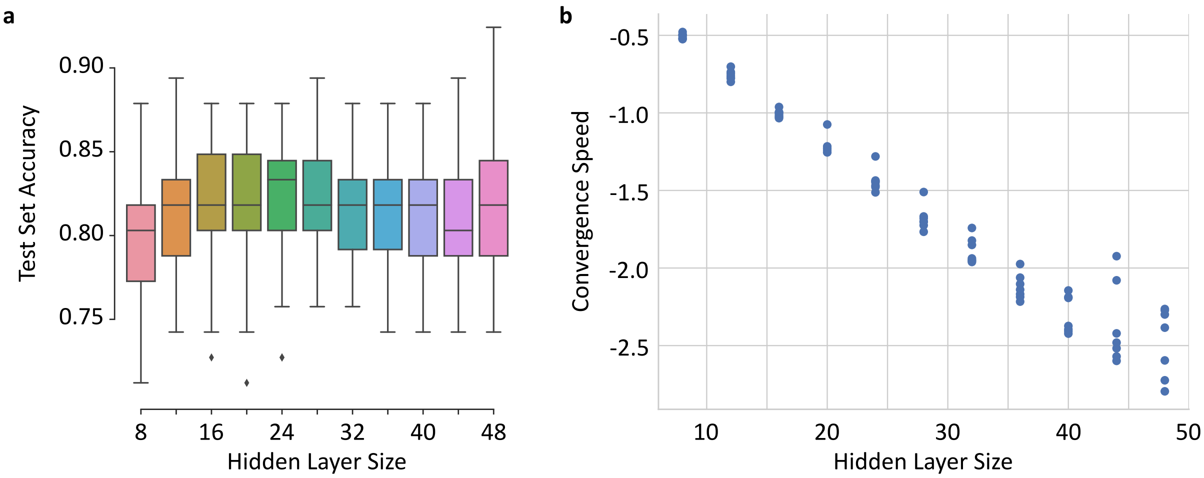

Figure S6. a: A scan over different hidden layer sizes for the multi-layer perceptron with one hidden layer shows a huge overlap of the box plots and thus does indicate a neglectable influence of the precise topology, except maybe for very small network sizes. **b:** Convergence speed of the loss function for multi-layer perceptrons with one hidden layer for sizes 8, 16, 32, 40, and 44 indicates that larger networks require fewer epochs to optimise the loss function. The convergence speed was determined as the exponent in an exponential fit to the loss function over the number of training epochs. To this end, for 10 repetitions, training was performed for 11 epochs without stopping condition with a 80 : 20 training to test split.

of the validation set. While the evaluation of 3 successive decoder iterations mitigates the initial fluctuations of the de-novo decoders validation accuracy, it leads to a long delay in a regime, where both decoders behave very similar, i.e., have very similar accuracy distributions in.

Therefore, a second approach proved equally effective, which requires a de-novo decoder accuracy of at least 80 % before switching. This value is chosen based on the decoder accuracy development shown in Fig. 6 such that the de-novo decoder's accuracy lies above the transition point at ≈ 75 %.

All trained decoders, PCA transformations, and feature vectors are stored for later re-use as pre-trained decoders.

Network Topology and Hyperparameter Selection

Based on the observation that very different decoders and topologies showed comparable performance (see Figure 3), especially the different multi-layer perceptron topologies, we did not perform extensive hyperparameter optimisation. Multi-layer perceptrons were selected due to their wide applicability and suitablilty for hardware optimisation. To complement Figure 3, for different multi-layer perceptrons with one hidden layer we performed 10 training runs with randomly selected samples in a 80 : 20 training to test split. The massively overlapping box plots in Figure **S6** **a** indicate that the size of the hidden layer for multi-layer perceptrons with one hidden layer doesn't seem to change the distribution of test set accuracies, as long as the network is not chosen too small. The investigation of test set accuracies gave no indication of overfitting and in the online case, test set accuracy was monitored to prevent overfitting by implementing early stopping.

Figure **S6** **b** shows the convergence speed of the loss function in a training setting without stopping over 11 epochs for differently sized networks. To determine the convergence speed, an exponential function $L(t) = a \cdot e^(\gamma t)$ was fitted to the loss function for 10 repetitions. We used the exponent γ as an indicator of the convergence speed measured in epochs. In Figure **S6** **b**, the exponents for all trained networks indicate that a larger size of the first hidden layer allows to complete network training in fewer epochs. Please note that the exponential function is neither a perfect fit, nor does this exponent γ directly relate to the absolute training speed, which depends a lot on the numerics and complexity of the topology and thus on the efficiency of the framework used.

We concluded that the exact topology of the multi-layer perceptron did not matter, if it is chosen to be at least 20. In regards to the tests performed for Figure 3 and the choice of 50 PCA components, we chose 50 as the maximum size of the hidden layer. Furthermore, we exploited faster training for extensive statistical analysis with Scikit-learn, using its vectorised numerics, by choosing a hidden network size of 50. While our software- and hardware-framework would orient themselves towards a lower FPGA-resource usage by choosing a smaller network size with 20 hidden neurons, as the pre-trained decoder doesn't need to be trained at runtime.

Supplementary Figure with Analysis for Monkey "K"

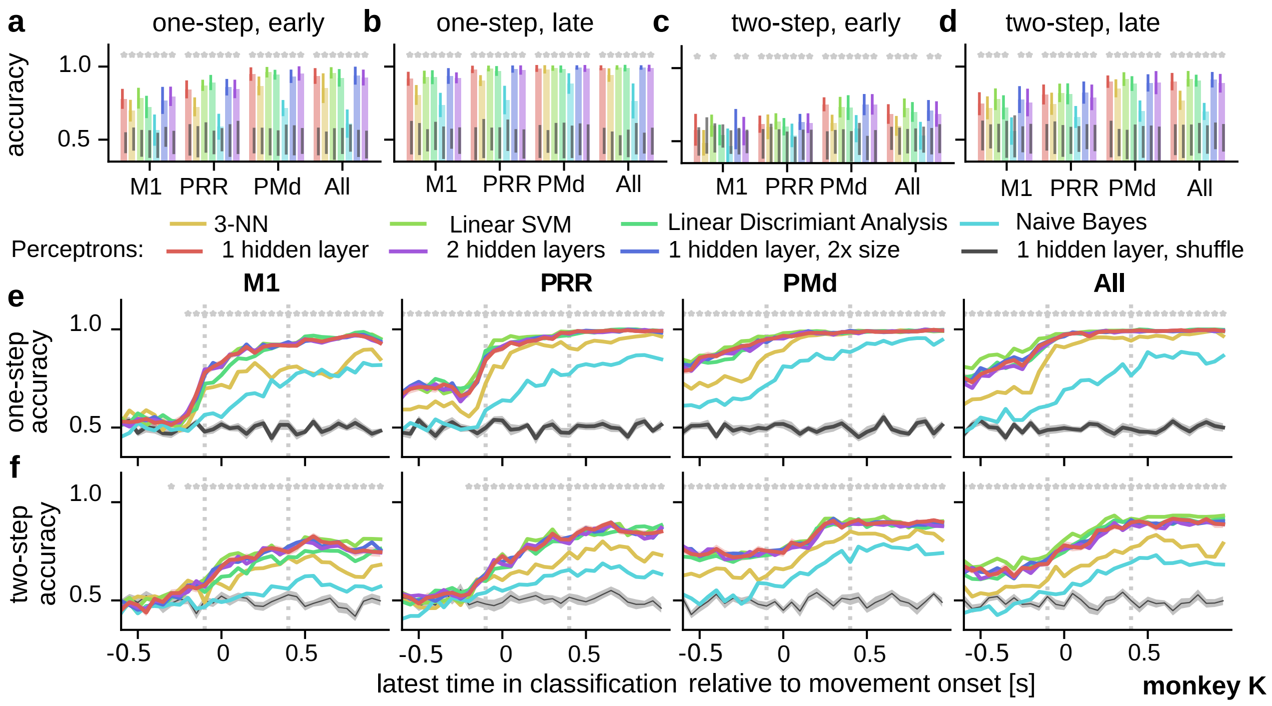

Figure S7. Offline decoding of planned action sequences from neural activities for monkey K, supplementing Figure 3. Comparison of the classification accuracy of various decoding algorithms for predicting the upcoming action in an one-step action sequence (close reach targets) before **(a)** and after **(b)** movement onset. Pale coloured bars and solid coloured error bars mark mean and standard deviation from a four times repeated, five-fold, stratified cross-validation for each classifier. Grey bars mark standard deviations for the same procedure with shuffled targets. Asterisks indicate significant difference (one-sided Mann-Whitney U-Test). Classifications were based on data from individual recordings from M1, PMd, PRR or from pooled activity (x-axis). **c, d:** Same as a and b, but for decoding the last action of a two-step action sequence. **e:** Decoding accuracy for an upcoming action (one step ahead in the sequence) by different classifiers for a 800 ms moving window ending at the time indicated on the x-axis. Classifications were based on data from individual recordings from M1 (1st panel), PRR (2nd panel), PMd (3rd panel) or from pooled data (4th panel). Grey curve represents the accuracy of the one-layer perceptron classifier with shuffled targets and asterisks a significant difference between shuffled and non-shuffled condition (Mann-Whitney U-Test). Shaded areas mark one SEM for the respective curves. Grey vertical lines mark the time points shown in panels a/c and b/d. **f:** Same as e, but for decoding the action plan of a two-step action sequence.

References

1. Gallego, J. A., Perich, M. G., Chowdhury, R. H., Solla, S. A. & Miller, L. E. Long-term stability of cortical population dynamics underlying consistent behavior. *Nat. neuroscience* **23**, 260–270 (2020).
2. Braun, J.-M., Carmo, R. A. R. F. & Fauth, M. Plan4act repository. https://github.com/plan4act (2021).
3. Curtin, R. R. *et al.* mlpack 3: a fast, flexible machine learning library. *J. Open Source Softw.* **3**, 726 (2018).
4. Huang, N.-S., Braun, J.-M., Larsen, J. C. & Manoonpong, P. A scalable echo state networks hardware generator for embedded systems using high-level synthesis. In *2019 8th Mediterranean Conference on Embedded Computing (MECO)*, 1–6 (IEEE, 2019).
5. Huang, N.-S., Chen, Y.-C., Larsen, J. C. & Manoonpong, P. AHEAD: Automatic holistic energy-aware design methodology for MLP neural network hardware generation in proactive BMI edge devices. *Energies* **13**, 2180 (2020).
6. Huang, N.-S., Larsen, J. C. & Manoonpong, P. Autobot for effective design space exploration and agile generation of RBFNN hardware accelerator in embedded real-time computing. In *2020 IEEE International Conference on Real-time Computing and Robotics (RCAR)*, 339–344 (IEEE, 2020).
7. Huang, N.-S., Braun, J.-M., Do Carmo, R. R., Larsen, J. C. & Manoonpong, P. End-to-end rapid FPGA prototyping for embedded proactive BMI control. In *2020 IEEE International Conference on Consumer Electronics-Taiwan (ICCE-Taiwan)*, 1–2 (IEEE, 2020).
8. Taylor, R. M. *et al.* Vrpn: a device-independent, network-transparent vr peripheral system. In *Proceedings of the ACM symposium on Virtual reality software and technology*, 55–61 (2001).

Supplementary Video SV1

Comparing reactive control (left panel) proactive control (right panel), the proactive control regime initiates all pending actions of the predicted action sequence at the time of decoding, whereas the reactive control regime will perform one action at a time. The resulting full-sequence time advantage of 13.25 s for proactive versus 21.22 s for reactive control (compare to Fig. 1c in the manuscript), is not based on the time of decoding, but on the completion of all controlled actuators.

Supplementary Video SV2

A presentation with walk through the full software & hardware framework, element by element. The presentation covers the full interaction from a neural stream recorded from a monkey in combination with the experiment task controller, over the hardware implementation of the decoder and proactive controller on a FPGA device, to a smart device, locally, as well as remotely, in the Living Lab Smart Home in Madrid. The latter is joined via a live video feed.
